# Bovine Teeth as Substitutes for Human Teeth in Dental Research: Ultrastructural and Radiographic Analysis

**DOI:** 10.1055/s-0045-1809032

**Published:** 2025-05-20

**Authors:** Mahmoud Al Ankily, Safaa Baz, Heba Mahmoud, Mahmoud M Bakr, Mohamed Shamel

**Affiliations:** 1 Oral Biology Department, Faculty of Dentistry, The British University in Egypt, Cairo, Egypt; 2 Oral Pathology Department, Faculty of Dentistry, The British University in Egypt, Cairo, Egypt; 3 General Dental Practice, School of Medicine and Dentistry, Griffith University, Gold Coast, Queensland, Australia

**Keywords:** bovine, scanning electron microscope, SEM, EDX, radiographic, enamel, dentin, cementum

## Abstract

**Objectives:**

Obtaining an alternative for human teeth in research remains challenging. The current study aimed to determine the validity of utilizing bovine teeth as a substitute for human teeth.

**Materials and Methods:**

Sound human maxillary premolars and bovine permanent lower central incisors were obtained. The human and bovine teeth were divided into groups ( *n* = 35) for scanning electron microscope (SEM) analysis alongside energy-dispersive X-ray spectroscopy (EDX) and optical radiographic density.

**Statistical Analysis:**

The data was statistically analyzed using the one-way analysis of variance along with a paired sample *t* -test, comparing the means of each two groups. The results were expressed as means ± standard deviations, and statistical significance was determined at an alpha level of 0.05.

**Results:**

SEM analysis of human and bovine samples in different hard tissues showed minor changes, mainly the human enamel had a smoother surface with distinct prism profiles, whereas the bovine dentin had larger and more widely separated dentinal tubules. EDX analysis revealed that the compositions of Ca and P, along with their Ca/P ratios in terms of enamel, dentin, and cementum, were comparable. For radiographic density, the findings revealed minor differences between human and bovine samples. No statistically significant differences among the studied groups were detected.

**Conclusion:**

This study revealed minor variations in structure, chemical composition, and radiographic density between human and bovine hard tissues, but without statistical significance, supporting the utilization of bovine teeth as a substitute for that of humans in dental research.

## Introduction

Human tooth specimens are preferred to be used for dental research, both *in situ* and *in vitro* , since they enable the study hypothesis to be evaluated in a more clinically relevant substrate. 1 Nevertheless, using human teeth has many restrictions since it is challenging to find enough healthy human teeth for laboratory testing. 2 Moreover, controlling the background and age of the human teeth that get collected is difficult, which could result in more variances in the study's outcome measures. As well as a greater understanding of the infection risk and ethical challenges. As a result, different substrates have been suggested and applied in dentistry research. 1

Dental research has used a variety of non-human tooth varieties as substrates for both *in situ* and *in vitro* studies. Bovine, 3 swine, 4 and shark teeth 5 are typical examples. The first factor to be considered when selecting potential animal replacements for human teeth is the properties of the hard tissues of the teeth. 6 Due to their availability and their substantial size in both the crown and root, numerous authors substituted bovine teeth for human teeth. 7 8 9 10

Bovine teeth could be used as an alternative dental substrate since they are simple to collect and can have their food, age, and other environmental parameters standardized, which reduces substrate discrepancies. 11 Moreover, there are no caries lesions or other defects on the broad surface of bovine teeth that could influence the outcome. 12

Even though bovine teeth are frequently utilized, some researchers are concerned about applying data from bovine teeth to human teeth due to differences in their chemistry and structure. 13 This study aimed to confirm the validity of substituting bovine teeth for human teeth in dental research and to discover these variations *in vitro* by comparing the results.

## Material and Methods

The research approval of this study was consented to by the Research and Ethics Committee of the Faculty of Dentistry, The British University in Egypt (Registration number FD BUE REC 24-002).

Utilizing G*Power (version 3.1.9.2) software, the recommended minimum sample size per group (human teeth and bovine teeth) was determined. Assuming a medium effect size ( *d* = 0.5), an alpha level of 0.05, and a power of 0.80, the calculation suggests 34 samples per group.

Permanent lower central incisors from bovine and human maxillary premolar teeth were used. The sound teeth of bovine, which were about 36 months old, were selected from a nearby slaughterhouse. The human teeth were healthy, free from caries or any pathology and were freshly erupted, from young males free from any medical conditions (12–15 years old) who has their teeth extracted for orthodontic purposes. Once the teeth were extracted, they were washed with distilled water to remove any residual blood, previously disinfected, and then prepared for sampling as follows.

### Scanning Electron Microscopy Analysis and Energy-Dispersive X-Ray Spectroscopy

Human teeth and bovine teeth were used for the scanning electron microscopy (SEM) analysis. Enamel specimens were obtained by separating the crowns from their roots at the cementoenamel junction. Then, low-speed handpiece holding discs were used to section the facial halves of the crowns under water coolant.

To obtain cementum samples, the cervical roots, measuring 5 mm in length, were divided mesiodistally, and the facial halves were utilized. The lingual halves were flattened on the outer surface to act as bases and were used as dentin specimens. The cementum, dentin, and enamel surfaces were unpolished.

The specimens were stored in distilled water at room temperature for 24 hours. They were then randomly divided into six groups ( *n* = 35 for each). The groups were as follows: group 1: human enamel (EH), group 2: bovine enamel (EB), group 3: human dentin (DH), group 4: bovine dentin (DB), group 5: human cementum (CH), and group 6: bovine cementum (CB).

Specimens from each group were imaged using SEM (Thermo Fisher Scientific Inc., Massachusetts, United States), Quattro S Felid Emission Gun, and Environmental SEM (FEG ESEM) at the Nanotechnology Research Center at the British University in Egypt to evaluate the surface topography. Additionally, specimens were assessed using energy-dispersive X-ray (EDX) analysis at two different points to investigate changes in the surface chemical composition of calcium (Ca) and phosphorus (P) elements. 14 15

### Optical Radiographic Density

Human premolars and bovine permanent incisors were stored in artificial saliva ( *n* = 35 for each). The crowns were transversally sectioned at the middle third into slices of 1.4 mm thick. All the slices were imaged with a direct digital sensor with an intraoral X-ray machine (HyperLight, Eighteenth, China), being exposed at 65 kV, 2.5 mA, for 0.1 seconds.

A special device was developed for the standardization of exposures, ensuring that the central X-ray beam was positioned perpendicular to the center of the sensor with a 9-cm focus-sensor distance. The tooth slices were placed so that they faced the X-rays in the center of the sensor. To prevent any potential effects on the radiodensity of the sections, the slice thickness, exposure period, target-sensor distance, kV, mA, and direction of the central X-ray beam were all standardized.

**Fig. 1:**
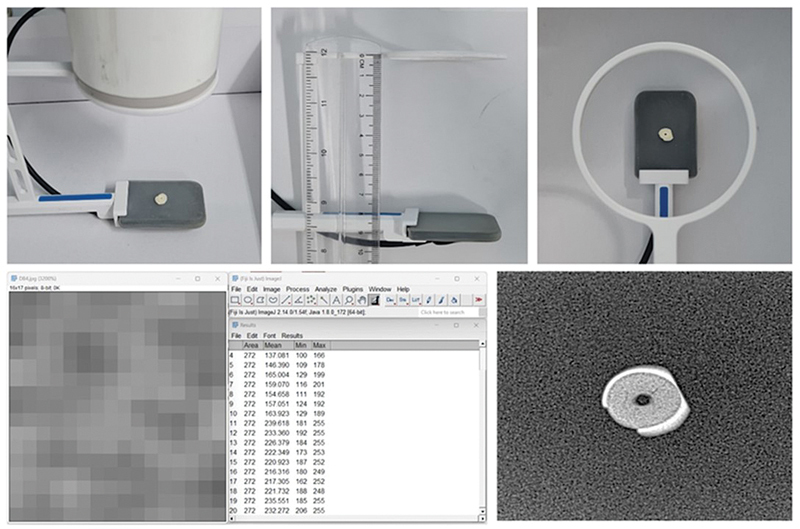
Setup process for radiographic imaging of tooth slices and analysis of gray values using Image J.

The digital radiograph of each slice was saved as jpg imaging. Then, the images were analyzed using Image J software (Fiji 1.54, United States) to measure the densities of the coronal enamel and dentin. The samples were analyzed for gray values according to their grayscale level, ranging from 0 to 255. The mean density values were used to calculate the enamel and dentin of each tooth 13 ( Fig. 1 ).

### Statistical Analysis

The statistical data analysis was performed using the one-way analysis of variance (ANOVA) along with a paired sample *t* -test using SPSS 22.0 statistical software (IBM Corp., United States). An ANOVA test was used to compare the mean density values of enamel and coronal dentin of human and bovine teeth to test the hypothesis that there are no statistically significant differences between these mean values. A paired sample *t* -test was performed to compare the means of human and bovine enamel, dentin, and cementum. A significance level of *α* = 0.05 was established. The results were expressed as means ± standard deviations, and statistical significance was determined based on the adjusted *p* -values.

## Results

### Morphological Structures of Human and Bovine Tooth Tissues

**Fig. 2:**
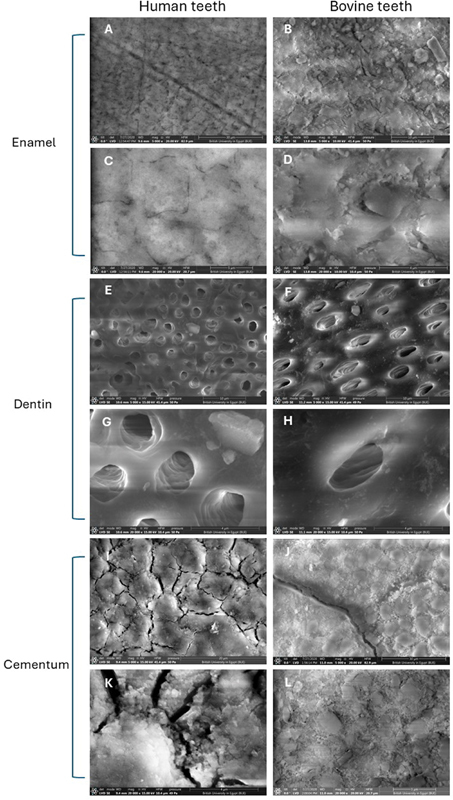
Scanning electron microscope (SEM0 analysis of healthy human and bovine teeth showing enamel ( **A** – **D** ), dentin ( **E** – **H** ), and cementum ( **I** – **L** ). Microphotographs were captured (A, B, E, F, I, and J) at ×5,000 and (C, D, G, H, K, and L) ×20,000 magnifications.

To evaluate the differences in enamel, dentin, and cementum structures between the human and bovine samples, teeth were examined by SEM. Regarding enamel, the surface micromorphology displayed an intact smooth surface of human enamel with some pits and microcracks. Prism profiles of variable distinctness resembled arcades, which were more obvious in humans than bovine enamel. Bovine enamel samples revealed slight structural and arrangement differences compared to human enamel ( Fig. 2A–D ).

The surface micromorphology of human and bovine dentin was quite similar. The human dentin displayed the typical SEM with a transverse section of dentinal tubules (DTs). The human dentin displayed a vertical arrangement of collagen fibers to the DT, interweaving into a mesh. The peritubular dentin (PD) and intertubular dentin revealed different degrees of mineralization. In comparison to human dentin, the surface characteristics of bovine dentin exhibited regular and patent DT that appeared to be more widely dispersed and had a larger diameter. In addition, there was a lower concentration in the number of DT per square millimeter. Moreover, the hypermineralization of PD was evident with uniform collagen fiber orientation ( Fig. 2E–H ).

For cementum, the surface micromorphology of human samples displayed rough surfaces with evident signs of wear and microcracks in large numbers. Meanwhile, the surface of bovine cementum exhibited less roughness with fewer cracks and/or signs of wear. Sharpey's fibers were hardly distinguished ( Fig. 2I–L ).

### Chemical Composition of Human and Bovine Tooth Tissues

**Fig. 3:**
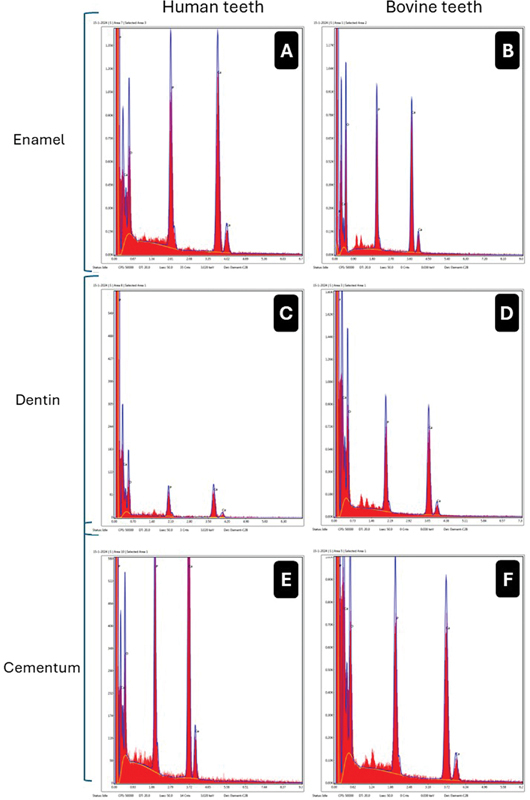
Energy-dispersive X-ray (EDX) spectra at two different points of healthy human and bovine teeth showing: enamel ( **A** , **B** ), dentin ( **C** , **D** ), and cementum ( **E** , **F** ).

EDX spectroscopy was used to determine the relative atomic surface concentration of elements in different study groups ( Fig. 3 ). The average weight percentages (wt%) of Ca and P in human and bovine samples, their corresponding ratios between Ca and P, along with average Ca/P ratio values for each group, were provided ( Table 1 ). Generally, there were differences in the elemental composition between human and bovine samples, with a higher average Ca/P ratio in human samples compared to the bovine ones.

**Table 1:** The average chemical composition of the enamel, dentin, and cementum of human and bovine teeth in different groups

	Human samples			Bovine samples		
	Element (average wt%)		Average Ca/P ratio	Element (average wt%)		Average Ca/P ratio
	Ca	P		Ca	P	
Enamel	37.38 ± 3.2	16.77 ± 0.7	2.23 ± 0.1	38.49 ± 1.3	17.09 ± 1.2	2.25 ± 0.2
Dentin	37.1 ± 1.9	14.49 ± 0.8	2.56 ± 0.1	32.65 ± 1.1	15.45 ± 1.8	2.15 ± 0.1
Cementum	40.39 ± 2.8	15.64 ± 2	2.6 ± 0.3	37.76 ± 3.7	14.25 ± 3.2	2.81 ± 0.5

Abbreviations: Ca, calcium; P, phosphorus.

For enamel, the human samples exhibited Ca percentages ranging between 32.34 and 40.64 wt%, P percentages from 15.85 to 17.55 wt%, and Ca/P ratios from 2.01 to 2.33. For bovine enamel, the Ca showed percentages from 36.13 to 40.45 wt%, P percentages from 15.85 to 19.97 wt%, and Ca/P ratios ranged from 2.03 to 2.45. The average Ca/P ratios of the human and bovine samples, 2.23 and 2.25, respectively, were quite similar.

In terms of dentin, the human samples showed Ca/P ratios of 2.01 to 2.33 with an average of 2.56. Percentages of P element ranged from 13.72 to 16.08 wt% (average of 14.49), and Ca percentages ranged from 32.75 to 42.51 wt% (average of 37.1). Ca percentages for bovine dentin ranged from 29.64 to 35.97 wt% (average of 32.65), P percentages from 12.32 to 17.28 wt% (average of 15.45), and Ca/P ratios from 1.59 to 2.79, with an average of 2.15.

The elemental composition of human cementum samples showed Ca weight percent ranging from 29.75 to 62.6%, P weight percent from 13.81 to 18.9%, and Ca/P ratios between 2.16 and 3.74. The bovine cementum samples exhibited Ca percentages from 30.24 to 41.03%, P percentages from 7.68 to 17.65%, and Ca/P ratios between 2.3 and 3.94. The average Ca/P ratios were higher in bovine cementum (Ca/P = 2.81) compared to human cementum (Ca/P = 2.6).

**Fig. 4:**
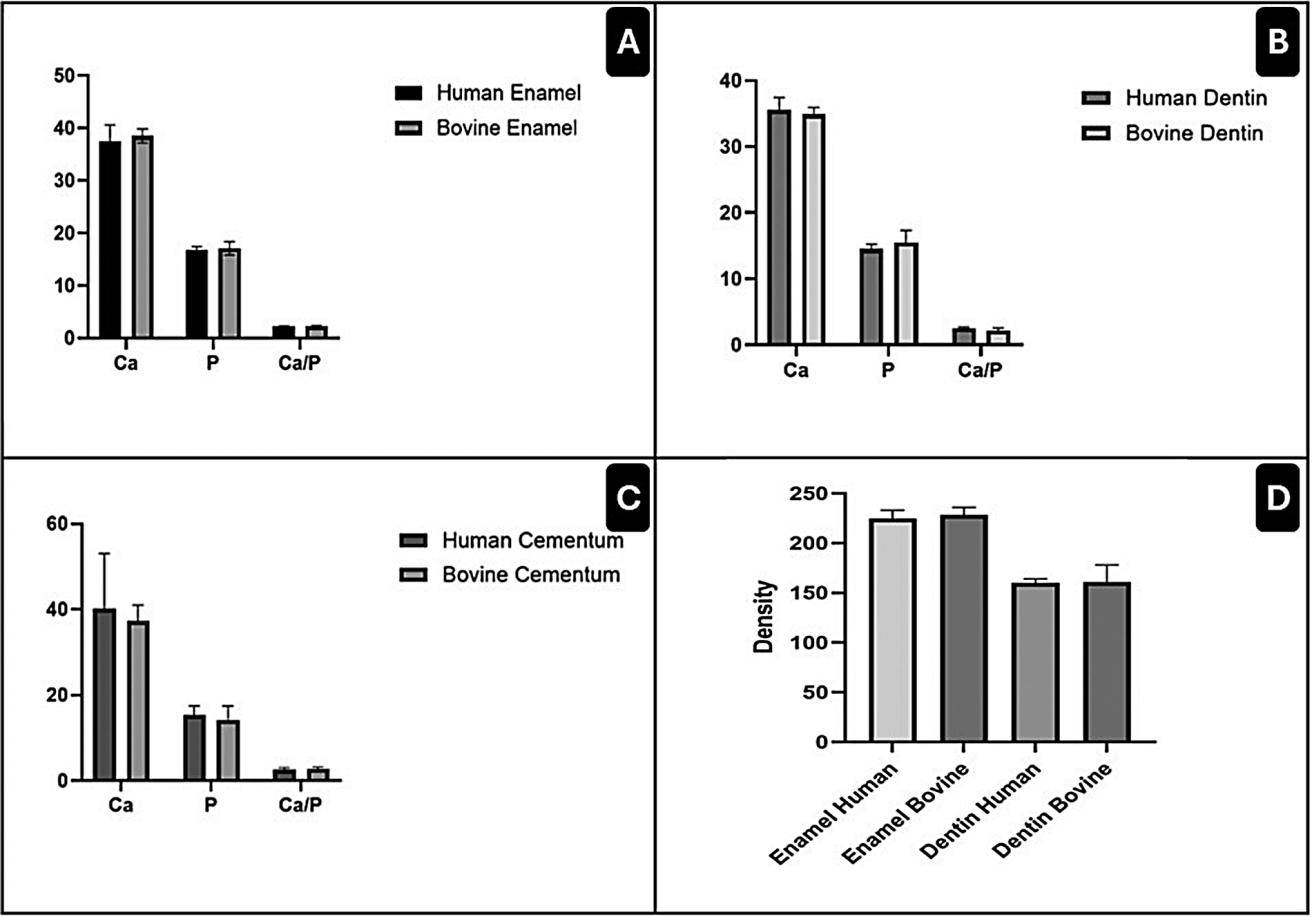
Compositional values of calcium (Ca), phosphorous (P), and Ca/P ratio, with error bars demonstrating standard deviations of human and bovine samples: enamel ( **A** ), dentin ( **B** ), and cementum ( **C** ). Optical mean density values of human and bovine enamel and dentin, with error bars indicating standard deviations ( **D** ).

Statistical analysis of Ca, P, and Ca/P ratios showed no significant differences between human and bovine samples for enamel. Similarly, there were no significant differences in the Ca and P components and their Ca/P ratios between human and bovine dentin. The comparison between human and bovine cementum regarding the Ca, P, and Ca/P ratios also revealed no significant differences ( Table 2 ) ( Fig. 4A–C ).

**Table 2:** Tukey's multiple comparisons test for Ca, P, and Ca/P ratio across human and bovine teeth in different groups

	Tukey's multiple comparisons test	Mean difference	95.00% CI of difference	Adjusted *p* -Value
Ca	Human enamel vs. bovine enamel	–1.110	–5.596 to 3.376	0.9801
	Human dentin vs. bovine dentin	1.547	–2.939 to 6.033	0.9191
	Human cementum vs. bovine cementum	2.928	–1.558 to 7.414	0.4166
P	Human enamel vs. bovine enamel	–0.3210	–4.807 to 4.165	> 0.9999
	Human dentin vs. bovine dentin	–0.9620	–5.448 to 3.524	0.9895
	Human cementum vs. bovine cementum	1.188	–3.298 to 5.674	0.9731
Ca/P	Human enamel vs. bovine enamel	–0.03600	–4.522 to 4.450	> 0.9999
	Human dentin vs. bovine dentin	0.4040	–4.082 to 4.890	0.9998
	Human cementum vs. bovine cementum	–0.1500	–4.636 to 4.336	> 0.9999

Abbreviations: Ca, calcium; CI, confidence interval; P, phosphorus.

### Optical Radiographic Density of Human and Bovine Tooth Tissues

The digital radiographic image of each section was measured to assess the optical radiography in enamel and dentin across different samples. Table 3 lists the average optical radiographic density in human and bovine teeth.

**Table 3:** The average density of optical radiography in the enamel and dentin of human and bovine teeth in different groups

		Human samples				Bovine samples			
		Area	Min	Max	Mean	Area	Min	Max	Mean
Enamel	Average	272	184.2	251.8	224.64 ± 8.2	272	183.4	254	228.53 ± 7.4
Dentin	Average	272	122.7	194.6	159.74 ± 4.1	272	129.4	194.2	163.95 ± 17

In terms of enamel, the average minimum density of human samples was 184.2, while the maximum value was 251.8. In close similarity, the bovine density had a minimum average of 183.4 and a maximum average of 254. Overall, there were minimal differences in the radiographic profile between human and bovine enamel samples, with mean averages of approximately 224.64 and 228.53, respectively.

For the dentin samples, the human average optical density maximum was 159.74, with a minimum average of 122.7 and a maximal average of 194.6. Meanwhile, the bovine dentin displayed an average minimum density of 129.4 and an average maximum of 194.2, with a mean average of approximately 164.

Statistically, the optical radiographic density showed no significant differences between human and bovine enamel. Similarly, there were no significant differences between human and bovine dentin ( Table 4 ) ( Fig. 4D ).

**Table 4:** Tukey's multiple comparisons test for optical radiographic density in the enamel and dentin of human and bovine teeth in different groups

	Tukey's multiple comparisons test	Mean difference	95.00% CI of difference	Adjusted *p* -Value
Enamel	Human enamel vs. bovine enamel	3.891	–8.616 to 16.40	0.8361
Dentin	Human dentin vs. bovine dentin	1.005	–11.50 to 13.51	0.9964

Abbreviation: CI, confidence interval.

## Discussion

Obtaining human teeth for research purposes remains challenging with ethical restrictions in dental studies. In view of the growing interest in finding an alternative for research and clinical practice, the current study was conducted aiming to determine whether it is valid to use bovine teeth in dental research instead of human teeth.

In this study, human premolars were chosen as they were extracted for orthodontic purposes. Meanwhile, the mandibular incisors of bovine were selected because of their similarity in size and shape to that of humans, besides the ease of extraction without surgery. Additionally, the occlusal surface of bovine molars has very little enamel exposed for microscopic examinations, which makes the anatomy of the teeth challenging for SEM research. Moreover, because the upper incisors of bovine appear as a compact connective tissue bulge covered in highly cornified, stratified pavement epithelium, they are also not available for use in dental research. 16

The purpose of this study was to investigate bovine teeth, which are readily available and do not require specific breeding or animal sacrifice to be used as an *in vitro* substitute for human teeth.

In this work, by SEM, the surface micromorphology displayed an intact smooth surface of human enamel with some pits and microcracks. Prism profiles of variable distinctness resembled arcades, which were more obvious in human than bovine enamel. Bovine enamel samples revealed slight structural and arrangement differences compared to that of human enamel. This could be attributed to the impact of the dietary type on development and orientation of the enamel prism, according to phylogenetic investigations. 17 Recently, a study denoted that bovine enamel prisms have an oval and narrow shape. 18

Consistent with the current study, the findings of Wang et al compared variations in the arrangement of prisms and interprismatic area between teeth from humans and bovines. They attributed it to the speedy development of bovine teeth during their formation and growth, both before and after they erupt, forming large crystal grains and lattice defects. 19

Regarding SEM analysis of dentin, the surface micromorphology of human and bovine samples exhibited almost similar features. However, the bovine DT differed structurally from the human DT, showing tubular structures with lower concentrations and greater diameters that were widely separated. This might be related to the rapid development of bovine teeth during their formation.

In the same context, a study performed by Lopes et al revealed that the diameter of the tubules in the superficial and middle layers of bovine dentin was notably larger compared to the tubule diameter in the superficial and middle layers of human dentin. However, no differences had existed between bovine and human DT diameter in the deeper dentin. 20 Additionally, other studies compared the application of bonds or adhesive materials to human and bovine dentin. Their results revealed differences in behavior due to the possible differences in the dentinal structure. 21 22 Despite these minor variations, the available *in vitro* literature indicated that utilizing bovine teeth in bond strength evaluations produces results similar to those obtained from human teeth, for both enamel and dentin substrates. 2

In terms of cementum, the SEM micromorphology of human samples displayed a smooth surface with various degrees of roughness and microcracks. This could be returned to physiological processes influenced by age, diet, and oral health. Comparably, the bovine cementum surfaces are often rougher and more irregular with extensive microcracks and defects, which could be attributed to the continuous deposition and wear from their herbivorous diet due to the greater mechanical forces exerted. Additionally, the insertion sites of the Sharpey's fibers, which could be represented as mounds or dish-topped projections, were difficult to identify. Generally, these fibers are smaller in diameter and more numerous in human teeth in comparison with bovine teeth. 23

EDX analysis was used to determine the Ca and P element ratios for the enamel, dentin, and cementum of human and bovine teeth at two distinct intact points. Then, the Ca/P ratios were calculated, and statistical analysis was carried out. Precise knowledge of elemental structure and chemical composition is an inevitable requirement to replace human teeth with animal teeth as an alternative for *in vitro* and *in situ* research. 24 EDX analysis was in alignment with the SEM results. It confirmed the close similarity between human and bovine teeth.

The EDX spectra of human enamel samples showed that the percentage of Ca ions were more than twice that of P ions. Consistently, the study of Sarna-Boś et al analyzed the chemical composition of human enamel. They found that Ca and P were the dominant elements, demonstrating high mineralization of the enamel. 25

The bovine enamel in this work showed close Ca and P weight percents to human levels, with no significant statistical differences. Additionally, the average Ca/P ratio of bovine and human enamel was calculated as 2.25 and 2.23, respectively, without significant statistical difference as well. Accordingly, Möhring et al indicated that the chemical composition of bovine and human enamel was nearly identical, demonstrating that Ca/P ratios for human and bovine enamel were particularly high and the differences were not statistically significant. 26 Another study performed by Olek et al, to evaluate the chemical composition of human and bovine enamel showed a highly mineralized tissue, forming salts of hydroxyapatite crystals. The elemental composition of the enamel of these samples was highly similar. 16

Concerning dentin, the EDX analysis revealed that the average Ca/P ratio, P percentage, and Ca percentage of human samples were 2.56, 14.49, and 37.1 weight percent, respectively. In comparison, bovine dentin had an average Ca/P ratio of 2.15, an average P percentage of 15.45 wt%, and an average Ca percentage of 32.65 wt%. These results showed a slightly higher Ca percentage and Ca/P ratio of human dentin compared to bovine dentin. Despite these minor differences, the overall elemental composition is remarkably comparable. This aligns with the statistical results, indicating no significant variations in the Ca, P, and Ca/P ratios of dentin between human and bovine samples.

In agreement with the current findings, a recent study compared the elemental composition of human and bovine dentin. The results demonstrated the statistical insignificance of the P and Ca ratios between bovine and human dentin, denoting that they can be used interchangeably in dental research. 26 The current study validates the use of bovine teeth instead of human extracted teeth. This is of high significance not only for dental researchers but also for researchers from different health-related disciplines that utilize teeth in their experimental work. The results from the present study confirm the reliability of previous published research using bovine teeth as the test subjects. Furthermore, it allows future research to be conducted using larger sample sizes with a higher reliability of results due to the significantly easier process of obtaining bovine teeth when compared to human extracted teeth.

The EDX spectrum of human cementum samples showed a wide range of Ca wt% ranging from 29.75 to 62.6%, with an average of 40.39 wt%. The average P wt% was 15.64%, and the average Ca/P ratio was 2.6. By comparing to the bovine cementum samples the averages of Ca, P, and Ca/P ratios were 37.76, 14.24, and 2.81, respectively. The statistical results revealed no significance between human and bovine cementum tissues. According to these results, there was a close similarity in the chemical composition between both, which confirms the suggested hypothesis of this study. To the best of our knowledge, no studies in the available literature compared the bovine and human cementum using EDX analysis.

Regarding the radiographic densities results, there were no statistically significant differences statistically between human and bovine teeth. Accordingly, a study performed by Fonseca et al evaluated the radiodensity of enamel and dentin of human and bovine teeth. They concluded that human and bovine radiodensities were similar with respect to enamel and dentin. 6

On the contrary, another study concluded that bovine enamel exhibited a significantly higher radiodensity compared to human enamel. Conversely, the radiodensity of bovine coronal dentin was significantly lower than that of human coronal dentin, and bovine radicular dentin had lower radiodensity than human radicular dentin as well. These differences were not statistically significant. They contributed these results to the differences in the composition and mineral content of each type of tooth, as well as variations in dietary habits. 13 27

A study recommended caution when interpreting findings obtained from experiments that used bovine teeth in substitution for human teeth. However, the study investigated the differences between human and bovine teeth at a crystalline level, which is different from the scope of other studies including the present investigation. 28 The reliability of bovine teeth as a replacement method for human teeth remains unquestioned unless the research methods are investigating properties at a nanoscale. A recent systematic review reported that about two-thirds of the studies that investigated the use of bovine teeth in laboratory studies showed that they can replace human teeth in a number of experimental settings that investigate a wide range of physical, chemical, and morphological variables. 29

This study provided an extended comparative investigation of the human and bovine teeth by analyzing SEM, EDX, and optical radiographic density. The findings revealed minor differences in the morphological, structural, and radiographical properties of the analyzed tissues. Despite these differences, no significant differences had been detected between humans and bovines with respect to enamel, dentin, and cementum tissues. These findings strongly support the study hypothesis that there were no statistically significant differences between the mean values of enamel and coronal dentin of human and bovine teeth. The minor changes observed between bovine and human teeth were mainly related to the chemical composition and are not significant since human extracted teeth show similar variations in the chemical composition. 25 30 Furthermore, individual human variations as well as environmental factors in the oral cavity are related to congenital factors and dietary habits. 31

## Conclusion

In conclusion, the current study indicated that bovine teeth could serve as a substitute for human teeth, offering substantial implications in dental research with precise consideration. In the future, further studies should be conducted to confirm the precision of bovine teeth hard tissues in laboratory research and clinical applications.
