# Supplementary material for: Bovine Teeth as Substitutes for Human Teeth in Dental Research: Ultrastructural and Radiographic Analysis
Source: Eur J Dent. 2025 May 20;20(2):457–65. doi: 10.1055/s-0045-1809032 (PMC13287924; doi:10.1055/s-0045-1809032)
Supplement: Supplementary file 1 — Supplementary Material [file 10-1055-s-0045-1809032-s2514060.pdf]

FD BUE REC
The British University in Egypt
Faculty of Dentistry
Research Ethics Committee

Research Report

Candidate name: Mahmoud Al Ankily, Mohamed Shamel, Heba Mahmoud, Safaa El Baz

Degree: research paper

Department: Oral Biology

Research title: Bovine teeth validity as a substitute from human teeth in dental researches.

Status:

- ■ Initial review
- ☐ Continuing review
- ☐ Final Report

Upon reviewing the research with reference to the scientific research ethics charter, Ethics committee decision is to;

- ☐ Approved as submitted
- ☐ Approved with minor modifications as listed below
- ☐ Postpone the decision to obtain more information as listed below
- ☐ Disapprove the protocol for the reasons listed below.
- ■ Exempt from approval of research ethic committee as it does not fall under the umbrella of human subjects and exempt from continuing review.

Date: 10/1/2024
Modifications:
Information needed:
Reasons for disapproval:

Research Approval Number: 24-002

Head of REC:
Prof. Dr. /Dalia Ghalwash

FD BUE REC
IRB00012490
RHDIRB2020061401
